# Deciphering the Reactive Pathways of Competitive Reactions inside Carbon Nanotubes

**DOI:** 10.3390/nano13010008

**Published:** 2022-12-20

**Authors:** Tainah Dorina Marforio, Michele Tomasini, Andrea Bottoni, Francesco Zerbetto, Edoardo Jun Mattioli, Matteo Calvaresi

**Affiliations:** 1 Dipartimento di Chimica “Giacomo Ciamician”, Alma Mater Studiorum-Università di Bologna, Via Francesco Selmi 2, 40126 Bologna, Italy; 2 Center for Chemical Catalysis—C3, Alma Mater Studiorum—Università di Bologna, Via Selmi 2, 40126 Bologna, Italy

**Keywords:** nanoreactors, carbon nanotubes, nanoconfinement, nucleophilic substitution S_N_2, elimination reaction E2, catalysis

## Abstract

Nanoscale control of chemical reactivity, manipulation of reaction pathways, and ultimately driving the outcome of chemical reactions are quickly becoming reality. A variety of tools are concurring to establish such capability. The confinement of guest molecules inside nanoreactors, such as the hollow nanostructures of carbon nanotubes (CNTs), is a straightforward and highly fascinating approach. It mechanically hinders some molecular movements but also decreases the free energy of translation of the system with respect to that of a macroscopic solution. Here, we examined, at the quantum mechanics/molecular mechanics (QM/MM) level, the effect of confinement inside CNTs on nucleophilic substitution (S_N_2) and elimination (*syn*-E2 and *anti*-E2) using as a model system the reaction between ethyl chloride and chloride. Our results show that the three reaction mechanisms are kinetically and thermodynamically affected by the CNT host. The size of the nanoreactor, i.e., the CNT diameter, represents the key factor to control the energy profiles of the reactions. A careful analysis of the interactions between the CNTs and the reactive system allowed us to identify the driving force of the catalytic process. The electrostatic term controls the reaction kinetics in the S_N_2 and *syn*/*anti*-E2 reactions. The van der Waals interactions play an important role in the stabilization of the product of the elimination process.

## 1. Introduction

Host–guest chemistry is emerging as a novel way to manipulate reactivity by confining reactive systems (guests) inside containers (hosts). Reaction mechanisms are affected by confinement inside nanoscale reactors such as calixarenes, cucurbiturils, cyclodextrins, metal–organic frameworks (MOF), and zeolites [1]. Confinement acts on the potential energy surface (PES) topology, which is responsible for the kinetics (catalyzing or inhibiting reaction channels) but also changes the thermodynamics of the reaction because of the different interactions that the supramolecular host establishes with the reactants/products of the reaction [2,3,4,5]. The confinement inside a chemical host can have two major effects: the first occurs when the shape/volume of the host cavity (partly) restrains the guest system, and the second occurs when new chemical interactions between the host and guest are established.

Carbon nanotubes (CNTs) are nanosystems formed by trivalent carbon atoms covalently linked to form hollow cylindrical structures. The inside of the cylindrical shape allows them to host molecules [6,7]. CNTs are characterized by great chemical inertia. A crucial difference between CNTs and other nanocontainers is that their low reactivity and high stability make possible reactions under harsh conditions that would destroy other hosts. Experimentally, CNTs have already been used as containers for preparative chemical reactions [8,9,10,11,12,13,14,15,16,17,18,19].

For example, CNT nanoreactors were used to control the regioselectivity of aromatic halogenation reactions [10] and azide–alkyne cycloadditions [13] or to modulate the catalytic activity of metal or metal oxide nanoparticles encapsulated inside the cavity of the CNTs [8,11].

The spatial confinement of the reactant molecules inside the nanotube [20,21,22,23,24] drastically affects both the regioselectivity and kinetics of chemical reactions [8,9,10,11,12,13]. Many computational studies investigated how the molecular confinement inside CNTs affect chemical reactions [25,26,27,28,29,30,31,32,33,34,35,36,37,38,39,40,41,42,43].

We previously studied the reaction between methyl chloride and a chloride anion (the prototypical S_N_2 reaction) inside the confined environment of CNTs of different sizes [39]. Here, considering ethyl chloride as a reactant, we evaluated the effect of CNT confinement in competitive reactions.

**Scheme 1:**
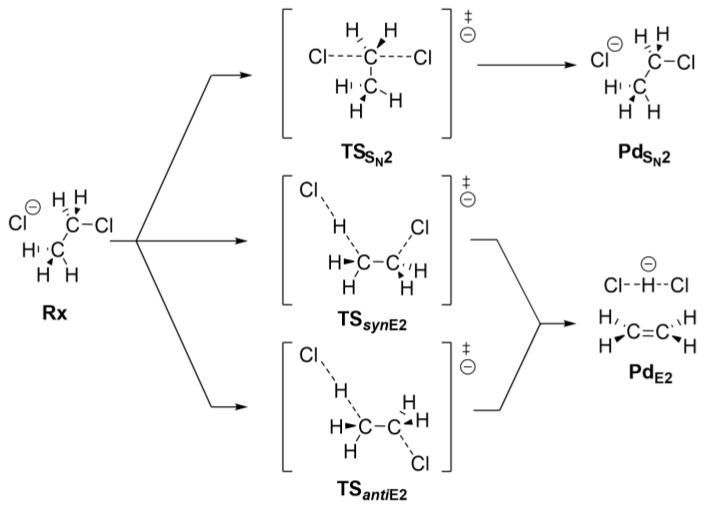
S_N_2, and *syn*- and *anti*-E2 reaction mechanisms.

Ethyl chloride can react with the chloride anion via either an S_N_2 or an E2 mechanism. Scheme 1 shows the S_N_2 mechanism (top) and the two pathways of the E2 mechanism, which occur via different stereochemical paths that depend on the *syn* (*syn*-E2) and *anti* (*anti*-E2) periplanar alignment of the leaving chloride anions and β-proton abstraction. Inside CNTs of different diameters, the requisite of periplanarity can be hard to satisfy and affect the possible reaction mechanisms. In this work, we used computational approaches to understand how the competitive S_N_2 and E2 mechanisms are affected by the CNT environment. An energy decomposition analysis was also performed to decipher the effects of the host–CNT environment on the reactivity of the *guest* systems.

## 2. Materials and Methods

The gas-phase reaction profiles were calculated at the DFT level with the M06-2X functional [44]. The functional was selected on the basis of previous studies [45,46]. The basis set used was 6-311++G(2df,2p) [47].

The coordinates of nanotubes 24 Å long were obtained with Nanotube Builder implemented in VMD [48]. CNT lengths greater than this value do not significantly affect the reactivity of guest systems [39].

The reaction profiles inside the CNTs were calculated using the ONIOM method available in Gaussian16 [49,50]. Mechanical embedding and electrostatic embedding were considered in the QM/MM calculations [49,51,52,53]. The high-level layer (i.e., the reactive guest system of ethyl chloride and chloride anion) was described at the M06-2X/6-311++G(2df,2p) level of theory. The low-level layer (i.e., the host CNTs) was described using the Universal Force Field (UFF) method [54]. Partial atomic charges calculated using the QEq scheme [55] were used to calculate electrostatic interactions. This methodology was effective to describe the energetics and the geometries of reactions of molecules confined inside CNTs [39], although some approximations are required to reduce the complexity of the system, such as the elimination of counter ions and solvent molecules inside the CNTs.

The structure’s minima and saddle points were fully optimized, and frequency calculations determined the nature of these critical points.

The effect of confinement on the reaction path ($\Delta E^{conf}$) was calculated as the difference between the QM/MM energy (reacting molecules in the CNTs) and the QM model system energy (reacting molecules in the gas phase).

Considering the scheme implemented in the ONIOM method, $\Delta E^{conf}$ can be decomposed as

(1) $$\Delta E^{conf}=\Delta E_{vdW}+\Delta E_{elect}+\Delta E_{strain\left(host\right)}+\Delta E_{strain\left(guest\right)}$$

where $\Delta E_{vdW}$ and $\Delta E_{elect}$ are the energy differences between host and guest of the van der Waals and electrostatic interactions (comprising both Coulomb interactions and the polarization effects due to the electrostatic embedding scheme). $\Delta E_{strain\left(host\right)}$ and $\Delta E_{strain\left(guest\right)}$ measure the energy strain of the host and the guest molecules induced upon the formation of the complex. These terms were calculated similarly to the Activation Strain Model (ASM) proposed by van Zeist and Bickelhaupt [56].

Within this formulation, the effect of confinement on the kinetics and thermodynamics of the S_N_2, *syn*-E2, and *anti*-E2 mechanisms is decomposed into a sum of contributions with a well-defined chemical meaning, providing a better comprehension of the role of the CNT cavity in the reactive system.

## 3. Results and Discussion

### 3.1. Potential Energy Surfaces for SN2, anti-E2, and syn-E2 Reactions in the Gas Phase

**Figure 1:**
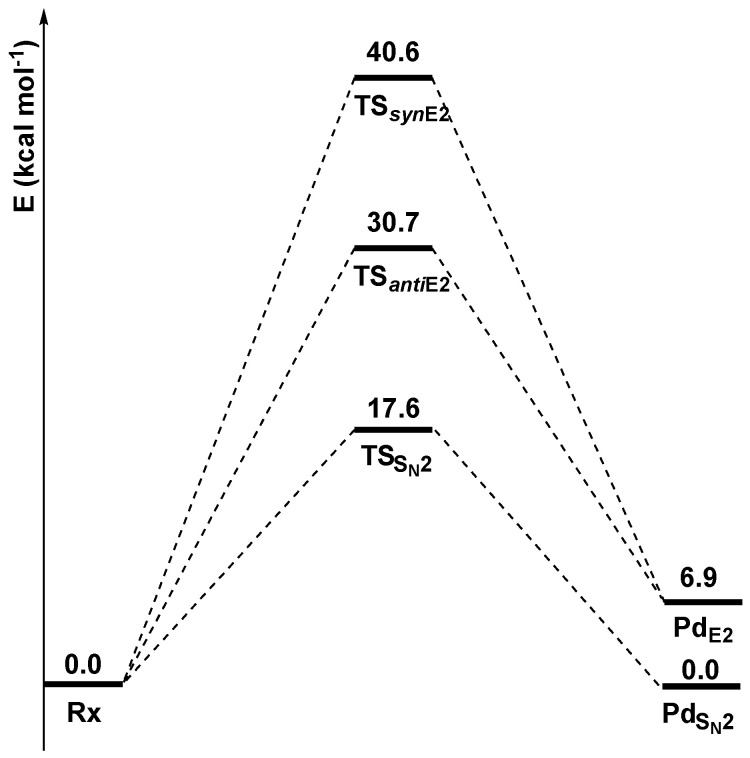
Gas-phase reaction profiles (kcal mol^−1^) of the S_N_2, *anti*-E_2_, and *syn*-E2 mechanisms computed at the M06-2X/6-311++G(2df,2p) level of theory.

Preliminary DFT calculations were carried out to calculate, at the M06-2X/6-311++G(2df,2p) level of theory, the stationary points of the potential energy surfaces of the three competitive mechanisms in the gas phase (Figure 1).

The approaching nucleophile Cl^−^ forms a preliminary complex (Rx) with ethyl chloride. From this complex, the system can follow three main paths: S_N_2, *syn*-E2, and *anti*-E2.

The S_N_2 mechanism, characterized by the lowest transition state energy (17.6 kcal mol^−1^), is the energetically favorite path and leads to a product (Pd_SN2_) isoenergetic with Rx, since the nucleophile and the leaving group are the same species (Cl^−^). Alternatively, the system can undergo an *anti*-E2 mechanism, with an activation barrier of 30.8 kcal mol^−1^, or a *syn*-E2 mechanism, with an activation barrier of 40.6 kcal mol^−1^. The two elimination pathways are endothermic, since Pd_E2_ is 6.9 kcal mol^−1^ higher in energy than Pd_SN2_, and lead to the formation of an ethylene molecule.

These results were benchmarked (Table 1) against “gold standard” calculations, reported in the literature at the Coupled Cluster CCSD and CCSD(T) levels, with infinite basis set extrapolation, with contributions of inner-shell correlation, scalar relativistic effects, and first-order spin-orbit coupling (CCSD(T)/CBS) [45].

### 3.2. Potential Energy Surfaces for SN2, anti-E2, and syn-E2 Reactions Confined in CNTs

**Figure 2:**
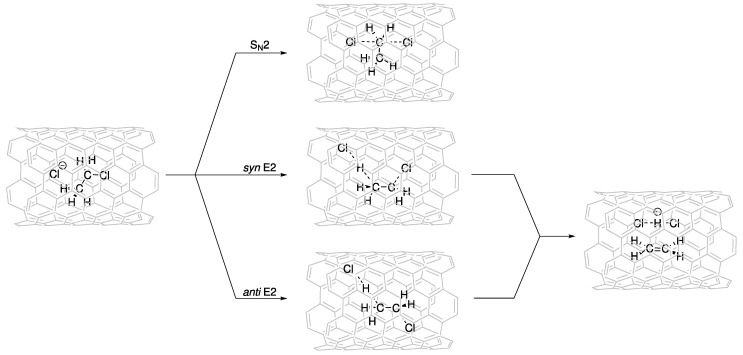
Schematic representation of S_N_2, and *syn-* and *anti*-E2 mechanisms inside CNTs.

We investigated the potential energy surfaces for S_N_2, and *syn-* and *anti*-E2 mechanisms inside CNTs of different diameters (Figure 2), namely, CNT(6,6), CNT(7,7), CNT(8,8), CNT(9,9), CNT(10,10), and CNT(12,12) (Figure S1).

The critical points corresponding to the reactant complex (Rx) and the S_N_2, and *syn*- and *anti*-E2 transition states (TSs) and products (Pds) were fully optimized inside the different CNTs. The activation ($\Delta E^{‡})$ and reaction (ΔE) energies of the reactions inside the different-diameter CNTs are reported in Table 2 and Table 3. The interactions between the CNT “nanoreactor” and the guest molecules were significantly large. Confinement could lead to either a decrease (catalysis) or increase (inhibition) in the activation energies (Table 2).

The interaction with the CNTs could also affect the thermodynamics of the reactions (Table 3) and, in particular, the E2 profile that was characterized by reactants and products with different geometries, which implies that locally, an energy difference is established among the isoenergetic reactants/products of the gas phase.

To better understand the effect of confinement in the substitution and elimination mechanisms, we discuss the two mechanisms separately in the next sections.

#### 3.2.1. Substitution Mechanism (S_N_2)

**Figure 3:**
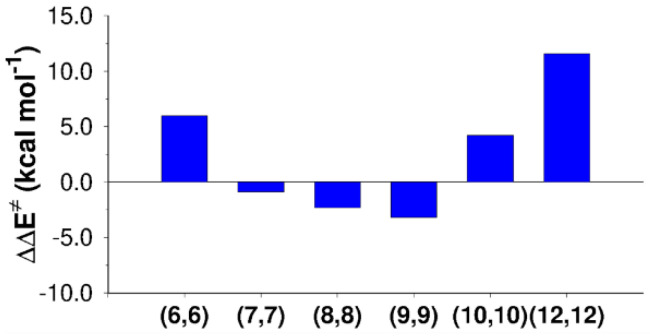
Activation energy variations $\Delta \Delta E^{‡}$ with respect to the gas-phase value ($\Delta E^{‡})$ of the S_N_2 reaction mechanism computed inside CNTs of different diameters.

The computed activation energies for the S_N_2 reaction inside the different tubes are reported in Table 2. A comparison with activation energy $\Delta E^{‡}$ in the gas phase (17.6 kcal mol^−1^) is shown in Figure 3, indicating the tendency of the reaction to be catalyzed or inhibited by the confinement within CNTs of different sizes.

**Figure 4:**
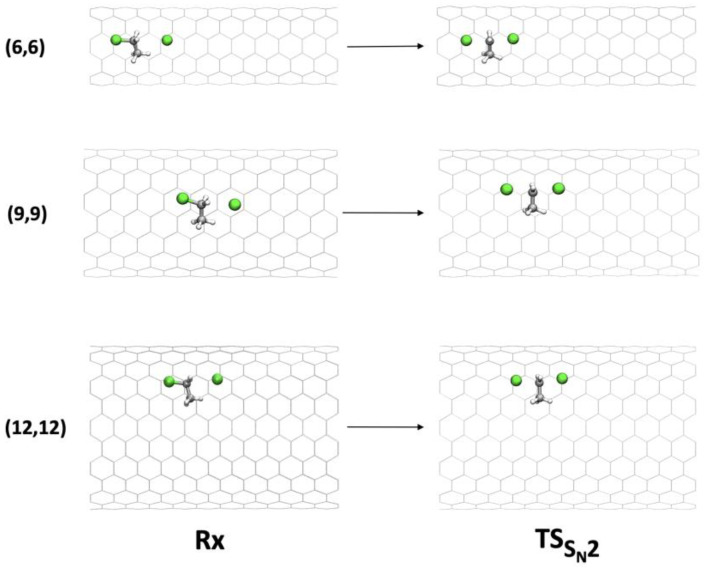
Geometries of Rx and TS of the S_N_2 mechanism inside (6,6), (9,9), and (12,12) CNTs.

Figure 3 shows that activation barrier $\Delta E^{‡}$ increased for the narrowest tube (CNT (6,6), $\Delta \Delta E^{‡}$ = 6.0 kcal mol^−1^) and for the larger tubes (CNT (10,10) and CNT (12,12), $\Delta \Delta E^{‡}$ of 4.2 and 11.5 kcal mol^−1^, respectively). On the contrary, the S_N_2 reaction was catalyzed by CNT(7,7), CNT(8,8), and CNT(9,9), showing decreases in the activation energy of 0.9, 2.3, and 3.2 kcal mol^−1^. Illustrative pictures are provided in Figure 4. An energy decomposition analysis of these terms ($\Delta \Delta E^{‡}$ in Table 4) was carried out to shed light on the contributions that affect the S_N_2 reaction confined inside CNTs.

The distortion of the host, Δ***E**_strain(host)_*, did not contribute to the modification of the kinetics of the S_N_2 reaction inside the CNTs, as it was close to 0 kcal mol^−1^ for all the tubes. This is not surprising because of the rigidity of the CNT walls. The energy term related to the distortion of the guest molecules, i.e., Δ***E**_strain(guest)_*, was also small. This effect can be associated with the rigidity of the guest system inside the CNT cavity. In general, the distortion of the reactive complex, imposed by the presence of the tube, is larger in Rx than in the transition state, because the geometry of the TS is less flexible (than the reactant complex); therefore, the imposed strain is lower. The higher destabilization of Rx compared with that of the TS gives a net stabilization energy.

In the largest tube (CNT(12,12)), the available volume was large enough to allow the reacting molecules to maintain the original arrangement observed in the gas-phase system; thus, the Δ***E**_strain(guest)_* term was zero.

When we analyze the chemical interactions occurring in the host/guest system, we can decompose it in two terms: the vdW term (Δ***E**_vdW_*) and the electrostatic term (Δ***E**_elect_*). Of course, the two terms act synergistically.

From the decomposition analysis, it appears that the electrostatic interaction governs the CNT effect on the S_N_2 kinetics. For CNT(6,6), CNT(10,10), and CNT(12,12), Δ***E**_elect_* was detrimental (5.5, 4.2, and 11.6 kcal mol^−1^), while for CNT(7,7), CNT(8,8), and CNT(9,9), the electrostatic interaction between the tube and the guest contributed to the catalyzing effect by −0.7, −1.9, and −4.0 kcal mol^−1^. This term takes into consideration the polarization induced on the tube by the reacting molecules.

#### 3.2.2. Elimination Mechanism (E2)

**Figure 5:**
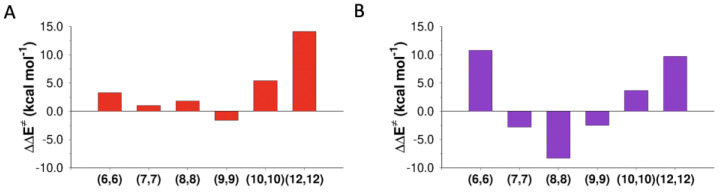
Activation energy variations with respect to the gas phase ($\Delta E^{‡})$ of the (**A**) *anti*-E2 and (**B**) the *syn*-E2 reactions inside CNTs of different diameters.

CNTs of different diameters have catalyzing/inhibiting effects also on the elimination mechanism. Figure 5 compares, with reference to the gas phase, the variation in the activation barrier of the two E2 mechanisms (*anti*-E2 and *syn*-E2) inside CNTs of different diameters.

**Figure 6:**
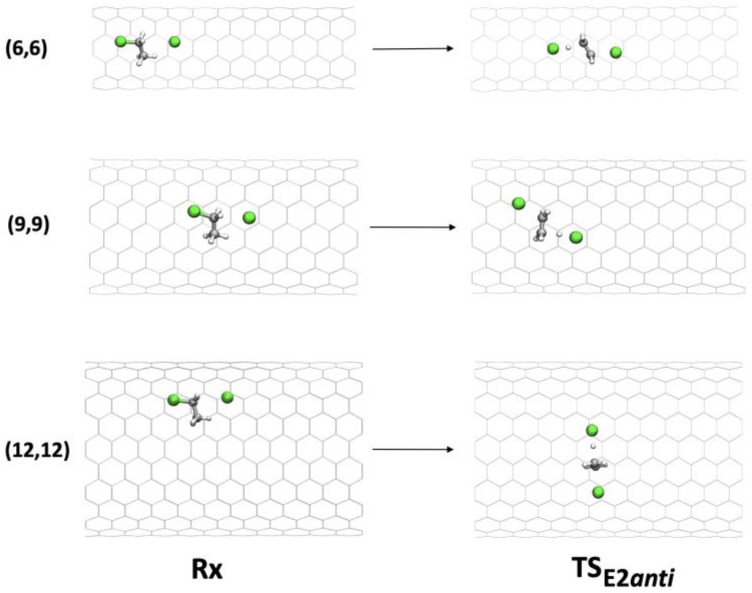
Geometries of Rx and TS in the *anti*-E2 mechanism inside (6,6), (9,9), and (12,12) CNTs.

The effect of confinement in the tubes is quite different for the two paths (*anti*-E2 and *syn*-E2 mechanisms). In fact, the *anti*-E2 mechanism is less sensitive to confinement. The main reason is the different orientation of the chlorine anion in the deprotonation step (Figure 6).

In the *anti*-E2 mechanism, the antiperiplanar geometry of the C-H and C-Cl bonds allows the TS to reorient inside the tube and to optimize its interaction with the tube walls (in particular with the chlorine atoms), compared with the *syn* disposition, which is blocked.

The result was that the effect of confinement on Rx and TS was similar for CNT(6,6), CNT(7,7), CNT(8,8), CNT(9,9), and CNT(10,10), and small variations were observed in the activation energies.

Two situations are particularly interesting: (i) inside CNT(9,9), the geometry of the TS was a perfect fit for the tube, and the interaction was stabilizing (catalysis); (ii) inside CNT(12,12), the tube was too large to interact at the same time with the two chlorines of the TS, and the reaction was slowed (inhibition).

**Figure 7:**
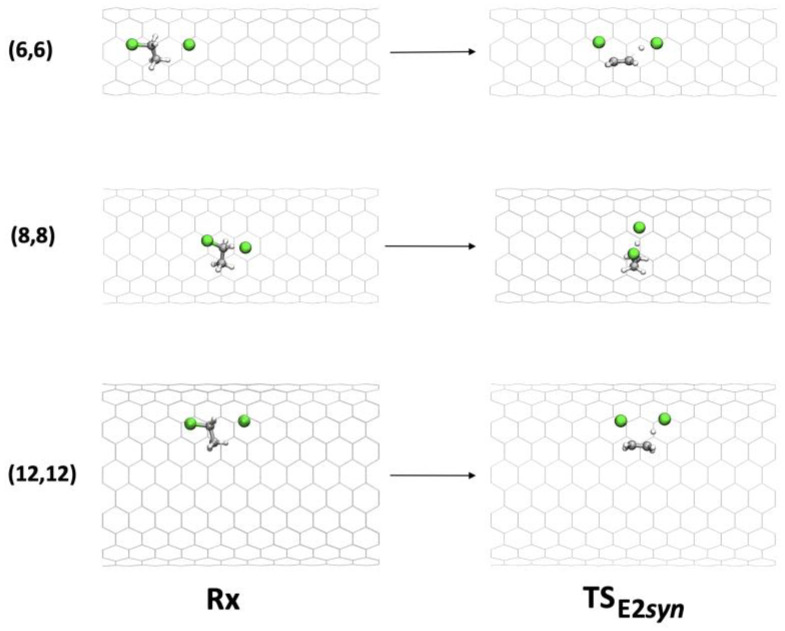
Geometries of Rx and TS in the *syn*-E2 mechanism inside (6,6), (8,8), and (12,12) CNTs.

On the other hand, the *syn*-E2 mechanism (Figure 7) has a well-defined conformation inside the CNT that allows both chlorine atoms to interact with the inner surface of the tube.

Inside CNT(6,6), in the TS, a distortion of the approach direction of the chlorine anion involved in deprotonation (C-H-Cl angles of 151° vs. 165° in the gas phase) was observed, with consequent destabilization. Inside CNT(7,7), CNT(8,8), and CNT(9,9), the chlorine anion was free enough to maintain its ideal angle of approach. The CNT walls fit well the TS structure, in particular in the case of CNT(8,8), in which the activation barrier was lowered by 8.3 kcal mol^−1^.

Again, for the larger CNT(10,10) and CNT(12,12), the stabilization of the TS was lower than that of Rx, and the reaction was globally inhibited.

The analysis of the energy contributions due to confinement inside the tubes (Table 5 and Table 6) showed that the electrostatic interactions between the CNTs and the guest system were once again responsible for the catalysis/inhibition of the *anti*-E2 and *syn*-E2 mechanisms inside the tubes. Particularly interesting is the value of Δ***E**_strain(guest)_* in the case of the *syn*-E2 mechanism inside CNT(6,6). A considerable value of 4.7 kcal mol^−1^ appeared and quantitatively described the distortion due to the lack of space for the TS inside the tube, which we previously described qualitatively.

Since the elimination mechanisms generate a product different from the reactant (in the S_N_2 mechanism, product and reactant are the same), the effect of the confinement on the reaction thermodynamics was also investigated.

**Figure 8:**
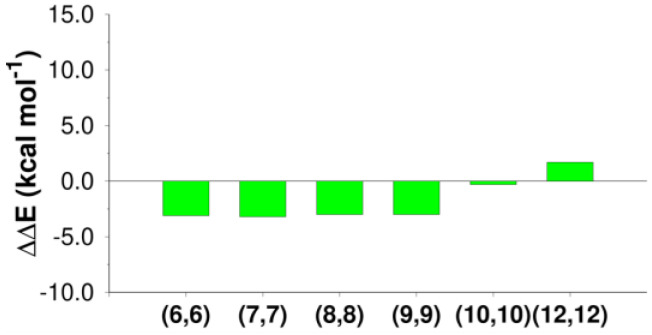
Local variation in the thermodynamics of the reaction with respect to the gas phase in the E2 reaction mechanism inside CNTs of different diameters.

As reported in Figure 8 and Table 7, CNTs provide the stabilization of the reaction product depending on the CNT size.

CNT(6,6), CNT(7,7), CNT(8,8), and CNT(9,9) stabilized the elimination product (ethylene) by ~3 kcal mol^−1^ (−3.1, −3.2, −3.0, and −3. kcal mol^−1^).

Inside CNT(10,10), stabilization was basically absent (−0.3 kcal mol^−1^), while CNT(12,12) destabilized the product (1.7 kcal mol^−1^).

The electrostatic term is always important, but in the present case, a crucial role is played by van der Waals interactions, since the conjugated system of the wall strongly interacts with the newly formed alkene via π-π interactions (these interactions, in the QM/MM model, are included in the van der Waals term). The use of multi-walled CNTs, which likely increases the E_vdW_ term, can potentially alter the products of the reaction, favoring the elimination channels.

## 4. Conclusions

A hybrid QM/MM method was used to describe the confinement effects on the competing S_N_2, *syn*-E2, and *anti*-E2 mechanisms of the reaction of ethyl chloride with chloride anion inside CNTs of different diameters. Confinement can have kinetic effects (either catalysis or inhibition), depending on the CNT diameter. The size of the nanoreactor represents the key factor to control the reaction profiles of the reactions. In general, when the geometry of the TS fits well with the size of the tube, the interaction is stabilizing (catalysis), similar to what happens with enzymes in their catalytic sites. When the tube is too narrow or too broad, the reactions are inhibited. In general, shape complementarity is a crucial factor for the interaction between carbon nanomaterials and molecules [57,58,59,60,61,62].

The selection of an appropriate substrate and of a CNT of the proper size may drive competitive reactions toward a target structure. For example, it may favor the elimination products against the substitution products by stabilizing the generated alkene or impeding the Walden inversion, typical of the S_N_2 channel.

The energy decomposition analysis of confinement effects in terms of strain of the guest and host systems, vdW, and electrostatic interactions demonstrated that the electrostatic term is the dominant contributor for both catalysis and inhibition. Nonetheless, in the case of elimination products, van der Waals contributions become important to modify the reaction thermodynamics.

To summarize, in this work, we show that the confinement of molecules inside CNTs can significantly alter the pathways of chemical reactions. The most straightforward use of these nanoreactors is to catalyze reactions, but they can also be used to inhibit the reactivity of molecules trapped inside the nanotube cavity. In this way, highly reactive molecules, such as polyynes or π-conjugated [63,64,65,66,67,68] polymers, can be confined and protected inside CNTs of suitable dimensions.

## Figures and Tables

**Table 1:** Critical point energies (kcal mol^−1^) of the S_N_2, and *syn*- and *anti*-E2 mechanisms. Energies were calculated with the M06-2X functional with the 6-311++G(2df,2p) basis set and reference CCSD(T)/CBS values obtained by Bento [45].

		S_N_2	*syn*-E2	*anti*-E2	E2
	Basis Set	$\Delta E^{‡}$	$\Delta E^{‡}$	$\Delta E^{‡}$	ΔE
**CCSD(T)**	CBS [45]	17.5	42.6	29.9	6.9
**M06-2X**	6-311++G(2df,2p)	17.6	40.6	30.7	6.9

**Table 2:** Activation energies (kcal mol^−1^) of the S_N_2, and *syn*- and *anti*-E2 reaction mechanisms inside CNT cavities.

		Gas Phase	(6,6)	(7,7)	(8,8)	(9,9)	(10,10)	(12,12)
**S_N_2**	$\Delta E^{‡}$	17.6	23.6	16.7	15.4	14.1	21.8	29.2
***syn*-E2**	$\Delta E^{‡}$	40.6	51.4	37.8	32.3	38.0	44.3	50.3
***anti*-E2**	$\Delta E^{‡}$	30.7	34.1	31.8	32.6	29.1	36.2	44.9

**Table 3:** Reaction energies (kcal mol^−1^) of the S_N_2, and *syn*- and *anti*-E2 reaction mechanisms inside CNT cavities.

		Gas Phase	(6,6)	(7,7)	(8,8)	(9,9)	(10,10)	(12,12)
**S_N_2**	ΔE	0.0	0.0	0.0	0.0	0.0	0.0	0.0
**E2**	ΔE	6.9	3.8	3.3	3.9	3.8	6.6	8.6

**Table 4:** Energy contributions (kcal mol^−1^) to the overall confinement effect on the S_N_2 reactions.

S_N_2						
	(6,6)	(7,7)	(8,8)	(9,9)	(10,10)	(12,12)
$\Delta E_{strain\left(host\right)}$	0.0	0.0	0.0	0.1	0.0	−0.1
$\Delta E_{strain\left(guest\right)}$	−0.2	−0.2	−0.7	−0.4	0.4	0.0
$\Delta E_{vdW}$	0.7	0.0	0.2	1.0	−1.3	−1.3
$\Delta E_{elect}$	5.5	−0.7	−1.9	−4.0	5.1	12.9
$\Delta \Delta E^{‡}$	6.0	−0.9	−2.4	−3.3	4.2	11.5

**Table 5:** Energy contributions (kcal mol^−1^) to the overall confinement effect on the *anti*-E2 kinetics.

*anti*-E2						
	(6,6)	(7,7)	(8,8)	(9,9)	(10,10)	(12,12)
$\Delta E_{strain\left(host\right)}$	−0.6	0.0	0.0	−0.1	−0.1	−0.3
$\Delta E_{strain\left(guest\right)}$	−0.7	0.2	−1.0	−0.2	0.1	0.0
$\Delta E_{vdW}$	−3.6	1.3	−1.7	0.8	−0.5	−2.4
$\Delta E_{elect}$	8.2	−0.5	4.5	−2.1	5.9	16.8
$\Delta \Delta E^{‡}$	3.3	1.0	1.8	−1.6	5.4	14.1

**Table 6:** Energy contributions (kcal mol^−1^) to the overall confinement effect on the *syn*-E2 kinetics.

*syn*-E2						
	(6,6)	(7,7)	(8,8)	(9,9)	(10,10)	(12,12)
$\Delta E_{strain\left(host\right)}$	0.1	0.0	0.0	0.2	−0.1	0.0
$\Delta E_{strain\left(guest\right)}$	4.7	−0.1	−1.0	0.2	0.2	0.2
$\Delta E_{vdW}$	0.5	−0.4	−1.9	−1.0	1.6	2.7
$\Delta E_{elect}$	5.5	−2.2	−5.4	−1.9	2.0	6.8
$\Delta \Delta E^{‡}$	10.8	−2.7	−8.3	−2.5	3.7	9.7

**Table 7:** Energy contributions (kcal mol^−1^) to the overall confinement effect on the E2 thermodynamics.

E2						
	(6,6)	(7,7)	(8,8)	(9,9)	(10,10)	(12,12)
$\Delta E_{strain\left(host\right)}$	−0.6	0.0	0.0	0.1	0.1	−0.3
$\Delta E_{strain\left(guest\right)}$	−0.4	1.2	2.2	0.5	0.8	0.7
$\Delta E_{vdW}$	−6.6	1.6	−1.7	−1.2	−1.9	−3.7
$\Delta E_{elect}$	4.5	−6.0	−3.5	−2.4	0.7	5.0
ΔΔE	−3.1	−3.2	−3.0	−3.0	−0.3	1.7

## Data Availability

The data presented in this study are available upon request from the corresponding authors.

## Supplementary Materials

The following supporting information can be downloaded at: https://www.mdpi.com/article/10.3390/nano13010008/s1, Figure S1: Accessible vdW diameters of CNTs.

## References

[1] Mouarrawis V., Plessius R., van der Vlugt J.I., Reek J.N.H. (2018). Confinement Effects in Catalysis Using Well-Defined Materials and Cages. Front. Chem..

[2] Chakraborty D., Chattararaj P.K. (2019). Bonding, Reactivity, and Dynamics in Confined Systems. J. Phys. Chem..

[3] Santiso E.E., George A.M., Turner C.H., Kostov M.K., Gubbins K.E., Buongiorno-Nardelli M., Sliwinska-Bartkowiak M. (2005). Adsorption and Catalysis: The Effect of Confinement on Chemical Reactions. Appl. Surf. Sci..

[4] Dai J., Zhang H., Dai J.J., Zhang H. (2021). Recent Advances in Catalytic Confinement Effect within Micro/Meso-Porous Crystalline Materials. Small.

[5] Yu Z., Ji N., Li X., Zhang R., Qiao Y., Xiong J., Liu J., Lu X., Yu Z., Ji N. (2022). Kinetics Driven by Hollow Nanoreactors: An Opportunity for Controllable Catalysis. Angew. Chem. Int. Ed..

[6] Khlobystov A.N., Britz D.A., Briggs G.A.D. (2005). Molecules in Carbon Nanotubes. Acc. Chem. Res..

[7] Britz D.A., Khlobystov A.N. (2006). Noncovalent Interactions of Molecules with Single Walled Carbon Nanotubes. Chem. Soc. Rev..

[8] Serp P., Castillejos E. (2010). Catalysis in Carbon Nanotubes. ChemCatChem.

[9] Khlobystov A.N. (2011). Carbon Nanotubes: From Nano Test Tube to Nano-Reactor. ACS Nano.

[10] Miners S.A., Rance G.A., Khlobystov A.N. (2013). Regioselective Control of Aromatic Halogenation Reactions in Carbon Nanotube Nanoreactors. Chem. Commun..

[11] Xiao J., Pan X., Guo S., Ren P., Bao X. (2015). Toward Fundamentals of Confined Catalysis in Carbon Nanotubes. J. Am. Chem. Soc..

[12] Miners S.A., Rance G.A., Khlobystov A.N. (2016). Chemical Reactions Confined within Carbon Nanotubes. Chem. Soc. Rev..

[13] Miners S.A., Fay M.W., Baldoni M., Besley E., Khlobystov A.N., Rance G.A. (2019). Steric and Electronic Control of 1,3-Dipolar Cycloaddition Reactions in Carbon Nanotube Nanoreactors. J. Phys. Chem..

[14] Iglesias D., Melchionna M. (2019). Enter the Tubes: Carbon Nanotube Endohedral Catalysis. Catalysts.

[15] Solomonsz W.A., Rance G.A., Khlobystov A.N. (2014). Evaluating the Effects of Carbon Nanoreactor Diameter and Internal Structure on the Pathways of the Catalytic Hydrosilylation Reaction. Small.

[16] Solomonsz W.A., Rance G.A., Suyetin M., la Torre A., Bichoutskaia E., Khlobystov A.N. (2012). Controlling the Regioselectivity of the Hydrosilylation Reaction in Carbon Nanoreactors. Chem. A Eur. J..

[17] Rance G.A., Solomonsz W.A., Khlobystov A.N. (2013). Click Chemistry in Carbon Nanoreactors. Chem. Commun..

[18] Solomonsz W.A., Rance G.A., Harris B.J., Khlobystov A.N. (2013). Competitive Hydrosilylation in Carbon Nanoreactors: Probing the Effect of Nanoscale Confinement on Selectivity. Nanoscale.

[19] Rance G.A., Miners S.A., Chamberlain T.W., Khlobystov A.N. (2013). The Effect of Carbon Nanotubes on Chiral Chemical Reactions. Chem. Phys. Lett..

[20] Pan X., Bao X. (2011). The Effects of Confinement inside Carbon Nanotubes on Catalysis. Acc. Chem. Res..

[21] Kondratyuk P., Yates J.T. (2007). Molecular Views of Physical Adsorption inside and Outside of Single-Wall Carbon Nanotubes. Acc. Chem. Res..

[22] Alves I., Magalhães A.L. (2019). BN-Doped Graphene and Single-Walled Carbon Nanotubes for the Catalysis of S_N_2 Reactions: Insights from Density Functional Theory Modeling. J. Phys. Chem..

[23] Tavares I.S., Figueiredo C.F.B.R., Magalhães A.L. (2017). The Inner Cavity of a Carbon Nanotube as a Chemical Reactor: Effect of Geometry on the Catalysis of a Menshutkin SN_2_ Reaction. J. Phys. Chem..

[24] Kuznetsov V. (2020). Stereochemistry of Simple Molecules inside Nanotubes and Fullerenes: Unusual Behavior of Usual Systems. Molecules.

[25] Halls M.D., Schlegel H.B. (2002). Chemistry Inside Carbon Nanotubes: The Menshutkin SN_2_ Reaction. J. Phys. Chem..

[26] Halls M.D., Raghavachari K. (2005). Carbon Nanotube Inner Phase Chemistry: The Cl^−^ Exchange SN_2_ Reaction. Nano. Lett..

[27] Santiso E.E., George A.M., Gubbins K.E., Buongiorno Nardelli M. (2006). Effect of Confinement by Porous Carbons on the Unimolecular Decomposition of Formaldehyde. J. Chem. Phys..

[28] Lu T., Goldfield E.M., Gray S.K. (2008). Classical Trajectory Studies of the D + H_2_ → HD + H Reaction Confined in Carbon Nanotubes: Parallel Trajectories. J. Phys. Chem..

[29] Lu T., Goldfield E.M., Gray S.K. (2008). Chemical Reactivity within Carbon Nanotubes: A Quantum Mechanical Study of the D + H_2_ → HD + H Reaction. J. Phys. Chem..

[30] Trzaskowski B., Adamowicz L. (2009). Chloromethane and Dichloromethane Decompositions Inside Nanotubes as Models of Reactions in Confined Space. Theor. Chem. Acc..

[31] Wang L., Yi C., Zou H., Xu J., Xu W. (2010). Rearrangement and Thermal Decomposition of Nitromethane Confined Inside an Armchair (5, 5) Single-Walled Carbon Nanotube. Chem. Phys..

[32] Wang L., Xu J., Yi C., Zou H., Xu W. (2010). Theoretical Study on the Thermal Decomposition of Nitromethane Encapsulated Inside Single-Walled Carbon Nanotubes. J. Mol. Struct. Theochem..

[33] Lu T., Goldfield E.M., Gray S.K. (2010). Classical Trajectory Studies of the D + H_2_ → HD + H Reaction Confined in Carbon Nanotubes: Effects of Collisions with the Nanotube Walls. J. Phys. Chem..

[34] Feng H., Qian Z., Wang C., Chen C., Chen J. (2011). Tuning the Energy Barrier of Water Exchange Reactions on Al (III) by Interaction with the Single-Walled Carbon Nanotubes. Dalton Trans..

[35] Wang L., Yi C., Zou H., Gan H., Xu J., Xu W. (2011). Initial Reactions of Methyl-Nitramine Confined Inside Armchair (5, 5) Single-Walled Carbon Nanotube. J. Mol. Model..

[36] Wang L., Yi C., Zou H., Xu J., Xu W. (2011). On the Isomerization and Dissociation of Nitramide Encapsulated Inside an Armchair (5, 5) Single-Walled Carbon Nanotube. Mater. Chem. Phys..

[37] Wang W., Wang D., Zhang Y., Ji B., Tian A. (2011). Hydrogen Bond and Halogen Bond Inside the Carbon Nanotube. J. Chem. Phys..

[38] Ravinder P., Subramanian V. (2013). Thermo Neutral SN_2_ Reaction within Pristine and Stone-Wales Defective BNNTs and CNTs. J. Phys. Chem..

[39] Giacinto P., Bottoni A., Calvaresi M., Zerbetto F. (2014). Cl^(−)^ Exchange SN_2_ Reaction inside Carbon Nanotubes: C-H···π and Cl···π Interactions Govern the Course of the Reaction. J. Phys. Chem. C.

[40] Giacinto P., Zerbetto F., Bottoni A., Calvaresi M. (2016). CNT-Confinement Effects on the Menshutkin S_N_2 Reaction: The Role of Nonbonded Interactions. J. Chem. Theory Comput..

[41] Marforio T.D., Bottoni A., Giacinto P., Zerbetto F., Calvaresi M. (2017). Aromatic Bromination of N-Phenylacetamide Inside CNTs. Are CNTs Real Nanoreactors Controlling Regioselectivity and Kinetics? A QM/MM Investigation. J. Phys. Chem..

[42] Mejri A., Picaud F., el Khalifi M., Gharbi T., Tangour B. (2017). Controlling Activation Barrier by Carbon Nanotubes as Nano-Chemical. J. Mol. Model..

[43] Fedoseeva Y.V., Orekhov A.S., Chekhova G.N., Koroteev V.O., Kanygin M.A., Seovskiy B.V., Chuvilin A., Pontiroli D., Ricco M., Bulusheva L.G. (2017). Single-Walled Carbon Nanotube Reactor for Redox Transformation of Mercury Dichloride. ACS Nano.

[44] Zhao Y., Truhlar D.G. (2008). The M06 Suite of Density Functionals for Main Group Thermochemistry, Thermochemical Kinetics, Noncovalent Interactions, Excited States, and Transition Elements: Two New Functionals and Systematic Testing of Four M06-Class Functionals and 12 Other Functionals. Theor. Chem. Acc..

[45] Bento A.P., Sola M., Bickelhaupt F.M. (2008). E2 and S(N)_2_ Reactions of X^−^ + CH_3_CH_2_X (X = F, Cl); an Ab Initio and DFT Benchmark Study. J. Chem. Theory Comput..

[46] Zhao Y., Truhlar D.G. (2010). Density Functional Calculations of E2 and S_N_2 Reactions: Effects of the Choice of Density Functional, Basis Set, and Self-Consistent Iterations. J. Chem. Theory Comput..

[47] Frisch M.J., Pople J.A., Binkley J.S. (1984). Self-Consistent Molecular Orbital Methods Supplementary Functions for Gaussian Basis Sets. J. Chem. Phys..

[48] Humphrey W., Dalke A., Schulten K. (1996). VMD—Visual Molecular Dynamics. J. Mol. Graph..

[49] Dapprich S., Komáromi I., Byun K.S., Morokuma K., Frisch M.J. (1999). A New ONIOM Implementation in Gaussian Part I. The Calculation of Energies, Gradients, Vibrational Frequencies and Electric Field Derivatives. Comput. Theor. Chem..

[50] Frisch M.J., Trucks G.W., Schlegel H.B., Scuseria G.E., Robb M.A., Cheeseman J.R., Scalmani G., Barone V., Petersson G.A., Nakatsuji H. (2016). Gaussian16 Revision C.01.

[51] Vreven T., Byun K.S., Komáromi I., Dapprich S., Montgomery J.A., Morokuma K., Frisch M.J. (2006). Combining Quantum Mechanics Methods with Molecular Mechanics Methods in ONIOM. J. Chem. Theory Comput..

[52] Chung L.W., Sameera W.M.C., Ramozzi R., Page A.J., Hatanaka M., Petrova G.P., Harris T.V., Li X., Ke Z., Liu F. (2015). The ONIOM Method and Its Applications. Chem. Rev..

[53] Vreven T., Morokuma K., Farkas Ö., Schlegel H.B., Frisch M.J. (2003). Geometry Optimization with QM/MM, ONIOM and Other Combined Methods. I. Micro-Iterations and Constraints. J. Comput. Chem..

[54] Rappé A.K., Casewit C.J., Colwell K.S., Goddard W.A., Skiff W.M. (1992). UFF, a Full Periodic-Table Force-Field for Molecular Mechanics and Molecular-Dynamics Simulations. J. Am. Chem. Soc..

[55] Rappé A.K., Goddard W.A. (1991). Charge Equilibration for Molecular-Dynamics Simulations. J. Phys. Chem..

[56] Van Zeist W.J., Bickelhaupt F.M. (2010). The Activation Strain Model of Chemical Reactivity. Org. Biomol. Chem..

[57] Khaliha S., Marforio T.D., Kovtun A., Mantovani S., Bianchi A., Luisa Navacchia M., Zambianchi M., Bocchi L., Boulanger N., Iakunkov A. (2021). Defective Graphene Nanosheets for Drinking Water Purification: Adsorption Mechanism, Performance, and Recovery. FlatChem.

[58] Di Giosia M., Zerbetto F., Calvaresi M. (2021). Incorporation of Molecular Nanoparticles Inside Proteins: The Trojan Horse Approach in Theranostics. Acc. Mater. Res..

[59] Berto M., di Giosia M., Giordani M., Sensi M., Valle F., Alessandrini A., Menozzi C., Cantelli A., Gazzadi G.C., Zerbetto F. (2021). Green Fabrication of (6, 5) Carbon Nanotube/Protein Transistor Endowed with Specific Recognition. Adv. Electron. Mater..

[60] Di Giosia M., Marforio T.D., Cantelli A., Valle F., Zerbetto F., Su Q., Wang H., Calvaresi M. (2020). Inhibition of α-Chymotrypsin by Pristine Single-Wall Carbon Nanotubes: Clogging up the Active Site. J. Colloid Interface Sci..

[61] Di Giosia M., Valle F., Cantelli A., Bottoni A., Zerbetto F., Fasoli E., Calvaresi M. (2019). Identification and Preparation of Stable Water Dispersions of Protein—Carbon Nanotube Hybrids and Efficient Design of New Functional Materials. Carbon N. Y..

[62] Calvaresi M., Bottoni A., Zerbetto F. (2015). Thermodynamics of Binding between Proteins and Carbon Nanoparticles: The Case of C60@Lysozyme. J. Phys. Chem..

[63] Shi L., Rohringer P., Suenaga K., Niimi Y., Kotakoski J., Meyer J.C., Peterlik H., Wanko M., Cahangirov S., Rubio A. (2016). Confined Linear Carbon Chains as a Route to Bulk Carbyne. Nat. Mater..

[64] Miyaura K., Miyata Y., Thendie B., Yanagi K., Kitaura R., Yamamoto Y., Arai S., Kataura H., Shinohara H. (2018). Extended-Conjugation π-Electron Systems in Carbon Nanotubes. Sci. Rep..

[65] Nishide D., Dohi H., Wakabayashi T., Nishibori E., Aoyagi S., Ishida M., Kikuchi S., Kitaura R., Sugai T., Sakata M. (2006). Single-Wall Carbon Nanotubes Encaging Linear Chain C10H2 Polyyne Molecules Inside. Chem. Phys. Lett..

[66] Nishide D., Wakabayashi T., Sugai T., Kitaura R., Kataura H., Achiba Y., Shinohara H. (2007). Raman Spectroscopy of Size-Selected Linear Polyyne Molecules C 2nH_2_ (*n* = 4–6) Encapsulated in Single-Wall Carbon Nanotubes. J. Phys. Chem..

[67] Chang W., Liu F., Liu Y., Zhu T., Fang L., Li Q., Liu Y., Zhao X. (2021). Smallest Carbon Nanowires Made Easy: Long Linear Carbon Chains Confined inside Single-Walled Carbon Nanotubes. Carbon.

[68] Gao E., Li R., Baughman R.H. (2020). Predicted Confinement-Enhanced Stability and Extraordinary Mechanical Properties for Carbon Nanotube Wrapped Chains of Linear Carbon. ACS Nano.

