# Supplementary material for: Deciphering the Reactive Pathways of Competitive Reactions inside Carbon Nanotubes
Source: Nanomaterials (Basel). 2022 Dec 20;13(1):8. doi: 10.3390/nano13010008 (PMC9823513; doi:10.3390/nano13010008)
Supplement: Supplementary file 1 [file nanomaterials-13-00008-s001.zip › nanomaterials-2101535 - supplementary.pdf]

nanomaterials

MDPI

*Article*

# Deciphering the Reactive Pathways of Competitive Reactions inside Carbon Nanotubes

**Tainah Dorina Marforio [1,2], Michele Tomasini [1], Andrea Bottoni [1], Francesco Zerbetto [1], Edoardo Jun Mattioli [1,2,*] and Matteo Calvaresi [1,2,*]**

[1] Dipartimento di Chimica "Giacomo Ciamician", Alma Mater Studiorum-Università di Bologna, Via Francesco Selmi 2, 40126 Bologna, Italy
[2] Center for Chemical Catalysis—C3, Alma Mater Studiorum—Università di Bologna, Via Selmi 2, 40126 Bologna, Italy
* Correspondence: edoardojun.mattioli2@unibo.it (E.J.M.), matteo.calvaresi3@unibo.it (M.C.)

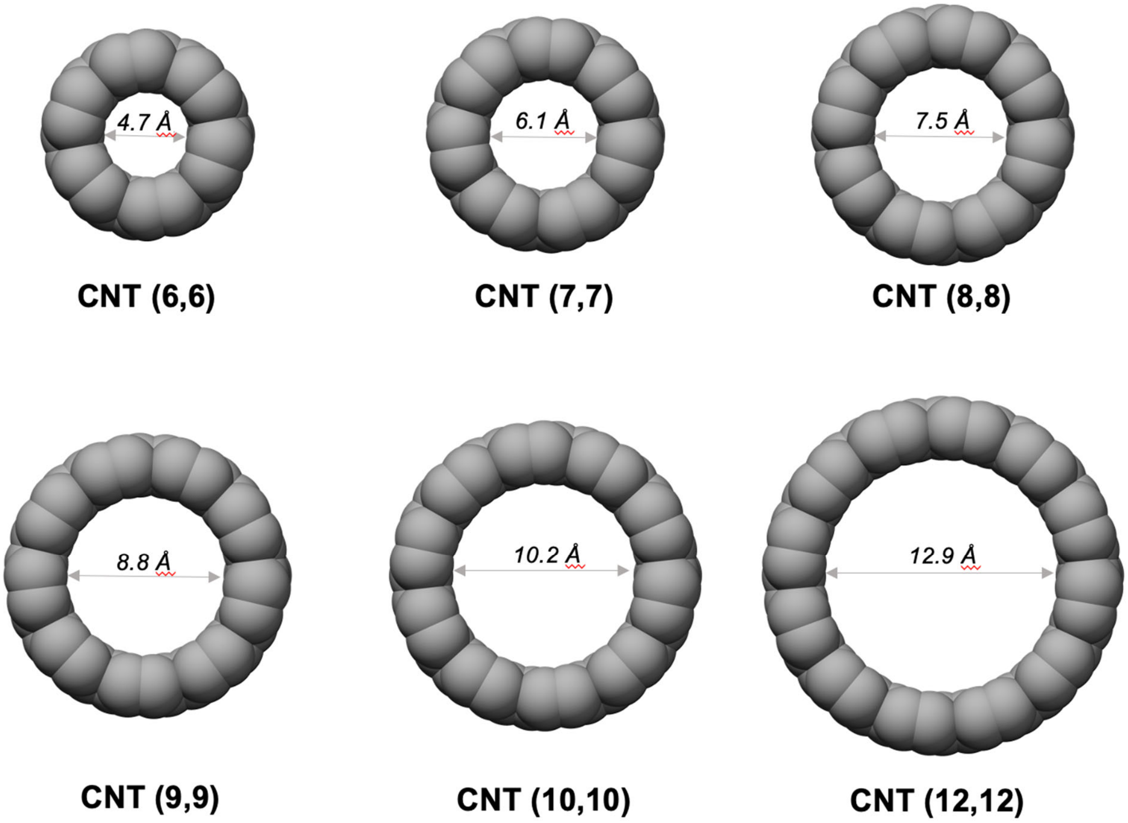


**Figure S1.** Accessible vdW diameters for CNT(6,6), CNT(7,7), CNT(8,8), CNT(9,9), CNT(10,10), CNT(12,12).
